# Supplementary material for: Supramolecular β-Cyclodextrin-Quercetin Based Metal–Organic Frameworks as an Efficient Antibiofilm and Antifungal Agent
Source: Molecules. 2023 Apr 23;28(9):3667. doi: 10.3390/molecules28093667 (PMC10179912; doi:10.3390/molecules28093667)
Supplement: Supplementary file 1 [file molecules-28-03667-s001.zip › molecules-2319177-supplementary.pdf]

Supramolecular β-Cyclodextrin-Quercetin Based Metal—Organic Frameworks as an Efficient Antibiofilm and Antifungal Agent

Rajaram Rajamohan [1,*], Chaitany Jayprakash Raorane [1], Seong-Cheol Kim [1,*], Mani Murali Krishnan [2] and Yong Rok Lee [1,*]

[1] School of Chemical Engineering, Yeungnam University, Gyeongsan 38541, Republic of Korea
[2] Department of Chemistry, Bannari Amman Institute of Technology, Sathyamangalam 638401, India
* Correspondence: rajmohanau@yu.ac.kr (R.R.); sckim07@ynu.ac.kr (S.-C.K.); yrlee@yu.ac.kr (Y.R.L.)

Experimental section and materials for biofilm

For *in-vitro* studies of QRC:β-CD-K MOFs, the *C. albicans* strain, namely DAY185 (fluconazole-resistant) is kindly offered by Prof. Jintae Lee, Yeungnam University, South Korea, and is originally acquired from the KCCM (South Korea) (http://www.kccm.or.kr/). Potato dextrose agar (PDA) or broth (PDB) is used to streak and sub-culture the *C. albicans* strain. Biofilm experimentations are directed overnight at an incubation temperature of 37°C utilizing a single colony inoculated in 15 ml of PDB media. By using at least two independent cultures all experiments are carried out. Clinical Laboratory Standards Institute (CLSI) for yeast (Clinical Laboratory Standards Institute, 2017), MIC is defined as the lowest concentration that inhibited cell growth. Briefly, freshly grown cells are diluted for the optimum size of inoculum for MICs. Cation-adjusted Mueller Hinton broth media are used in this study. For assessment of the antifungal efficacy of QRC:β-CD-K MOFs was performed using the well diffusion method. For the spread method, the total number of surviving fungi cells were determined by adding serial dilutions of the cells to agar plates. Fungi cells were cultured in a constant temperature incubator for 24 h. The total number of surviving cells were counted and % of cell survival was determined. Experiments are performed using at least three independent cultures.

Architecture of *C. albicans* biofilm

The Phenotypic differences and biofilm architecture of *C. albicans* DAY185 on LCS are examined using a previously explained protocol. Briefly, small pieces (0.5 cm x 0.5 cm) of nylon filter papers (0.45 μm) are placed in wells of 96-well plates comprising *C. albicans* grown in liquid media PDB and further treated by QRC:β-CD-K MOFs (0–50 μg/ml) for 24 h, 37 °C. Adhered *C. albicans* cells to the nylon filter surface are fixed by adding 40 μl of 1:1 mixture of formaldehyde (2%) and glutaraldehyde (2.5%) in each well. For the post-fixation and staining of cells osmium tetroxide: PBS (1:1) is used. A graded series of ethanol (50, 70, 90, 95, and 100%) is used for the dehydration of the sample. All samples are coated with gold for 100s followed by SEM images observed by SEM (S-4800 SEM, Hitachi, Tokyo, Japan) at an accelerating voltage of 10 kV.

Biofilm observations by confocal laser scanning microscopy

For the CLSM assay, single strain biofilms of *C. albicans* are produced in 96-well plates (with or without QRC:β-CD-K MOFs) at 37°C for 24 h without shaking. The free-floating cells are then discarded by rinsing with water two times, and biofilm cells attached to the surface of the wells are stained with CFDA-SE (carboxyfluorescein diacetate succinimidyl ester) (Invitrogen, Molecular Probes, Inc, Eugene, USA). The bottom of each well is then visualized using a 488 nm Ar laser (emission 500 to 550 nm) using a CLSM (Nikon, Tokyo, Japan). COMSTAT software is employed to determine the mean biofilm thicknesses (μm), biomass ($\mu m^3/\mu m^2$), and substratum coverages (%). Two independent samples are analyzed for each experiment, and more than 10 random spots are observed.

Characterization

Various techniques are used to analyze the MOFs, including FT-IR spectroscopy, proton NMR, FE-SEM with EDX spectroscopy, XRD, XPS, and DSC. FT-IR spectra are recorded with the Perkin Elmer Spectrum Two in transmittance mode within the ranges of 400.0 - 4000.0 cm^{-1}. 16 scans for the measurement at a resolution of 8 cm^{-1}. proton nuclear magnetic resonance (^1H NMR) spectroscopy (Bruker NMR spectrometer with 600.0 MHz). At a 10.0 kV accelerating voltage, FE-SEM, and EDX spectral analysis is carried out with the Hitachi S-4800. Powder XRD measurements are performed on a PANalytical X'Pert3 MRD diffractometer at 40.0 kV and 30 mA with monochromatized Cu K radiation (λ =1.54 Å). The 2θ range is 10° - 80° at a scan rate of 5° min^{-1} and a wavelength of 1.5405 Å. K-Alpha is used to generate XPS spectra (Thermo Scientific). The CasaXPS software S3 is used to deconvolve the high-resolution XPS spectra. The thermal behavior of the MOFs is performed using TA instruments, and the curves are of individual elements analyzed using the Universal V4.5A Program. The weight of each sample is around 3.5 mg and the temperature range for the measurement is about 40.0 – 250 °C under a dynamic nitrogen atmosphere (50 mL min−1) with increments of 10 °C/minute. All of the above instrument services are utilized at the core research support center (CRSC) for natural products and medical materials at Yeungnam University, South Korea.

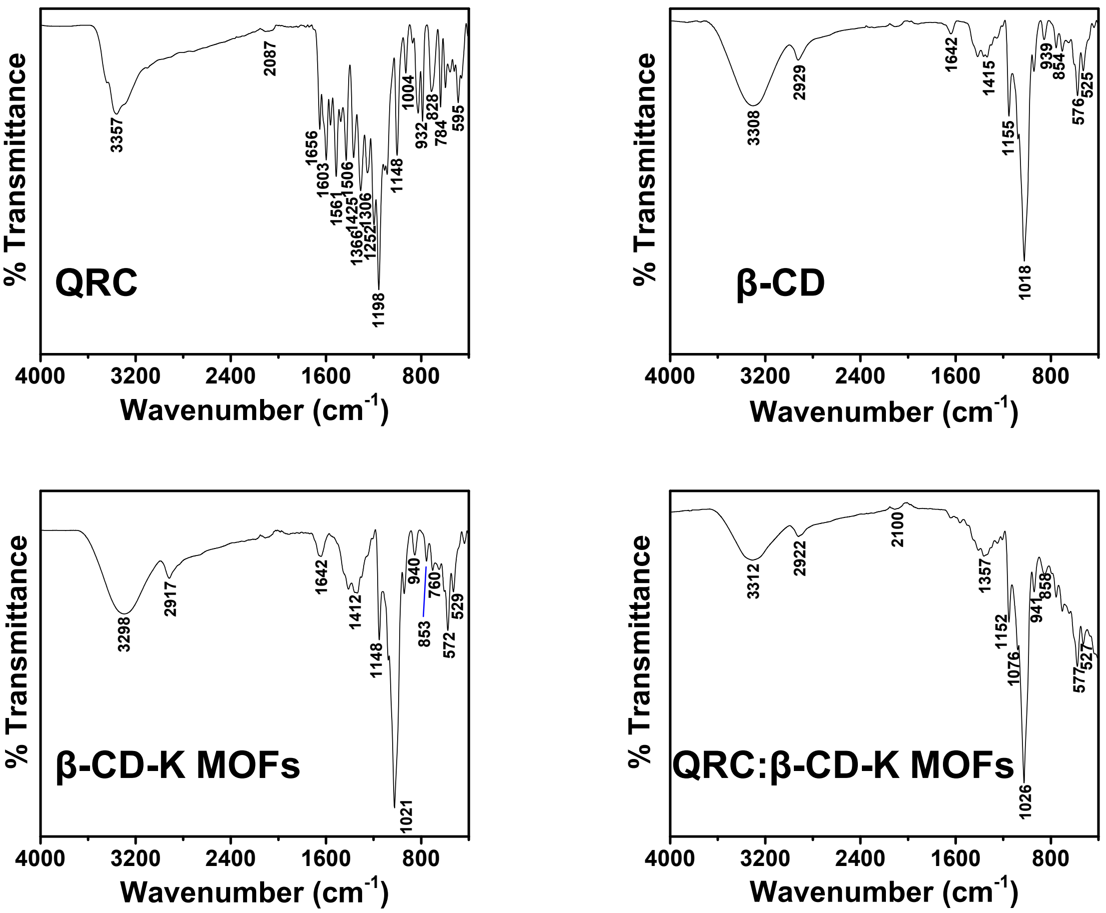

Figure S1. FT-IR spectra of QRC, β-CD, β-CD-K MOFs, and QRC:β-CD-K MOFs.

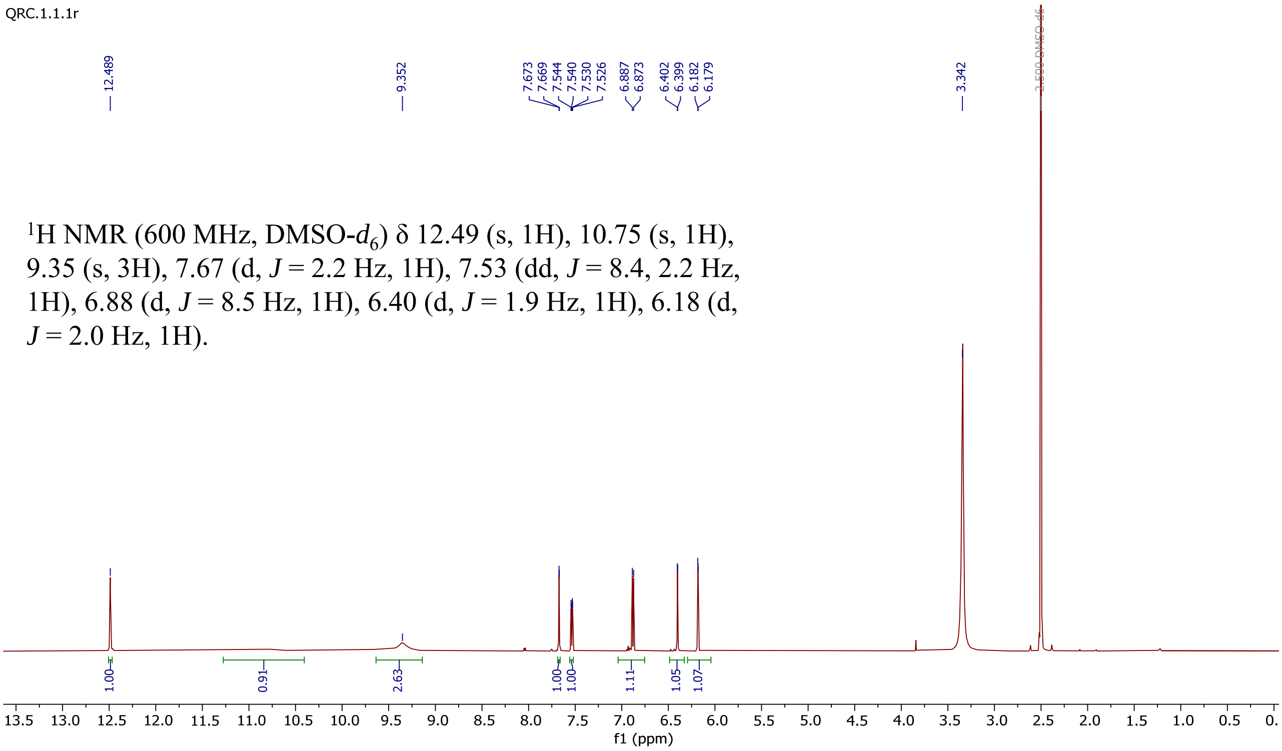

^{1}H NMR (600 MHz, DMSO-d_6) δ 12.49 (s, 1H), 10.75 (s, 1H), 9.35 (s, 3H), 7.67 (d, J = 2.2 Hz, 1H), 7.53 (dd, J = 8.4, 2.2 Hz, 1H), 6.88 (d, J = 8.5 Hz, 1H), 6.40 (d, J = 1.9 Hz, 1H), 6.18 (d, J = 2.0 Hz, 1H).

Figure S2. ^{1}H NMR spectra of QRC

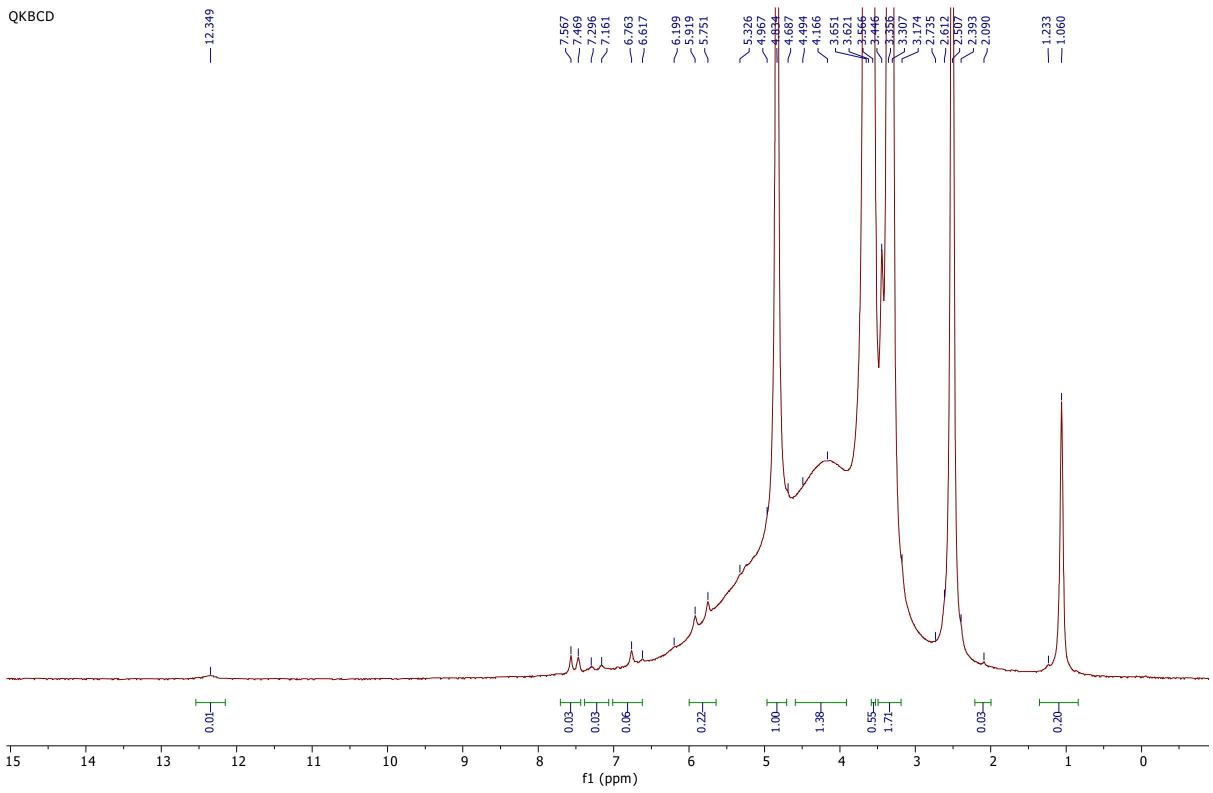

Figure S3. ^{1}H NMR spectra of QRC:β-CD-K MOFs.

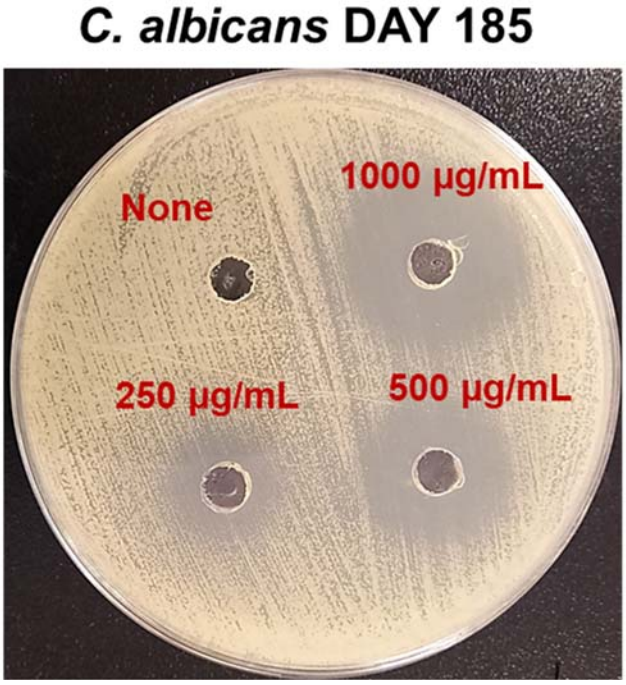

Figure S4. Dose dependent antifungal efficacy of QRC:β-CD-K MOFs.

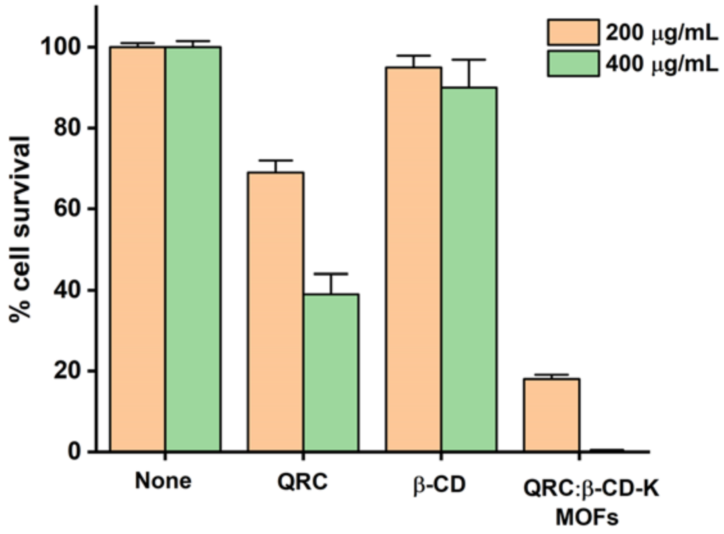

Figure S5. Percentage of *C. albicans* DAY 185 cell survival against different concentrations of QRC, β-CD and QRC:β-CD-K MOFs.

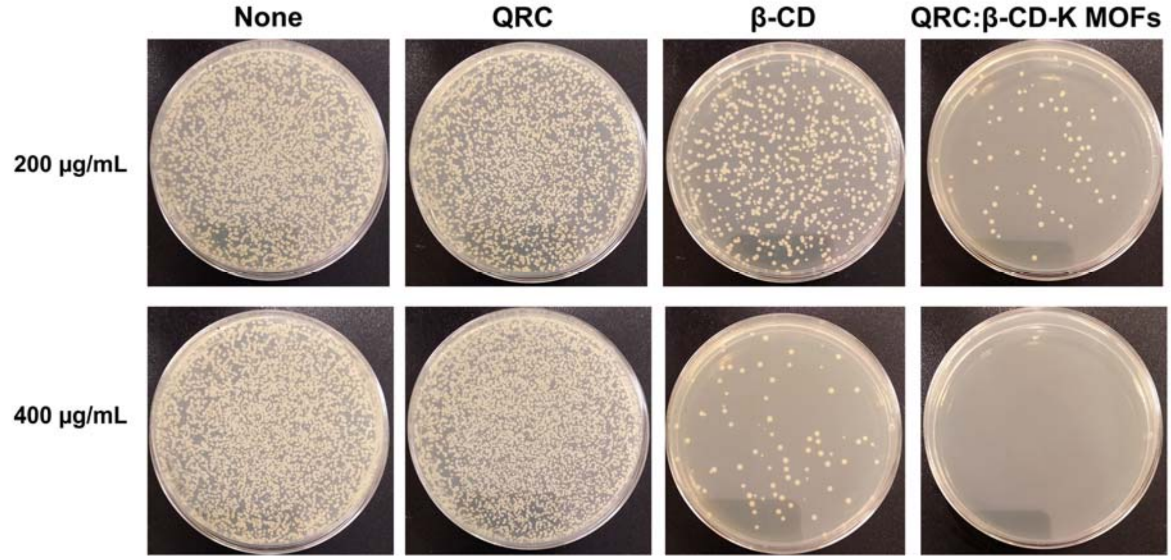

Figure S6. Representative images of spread plate method to characterize live *C. albicans* cells against different concentrations of QRC, β-CD and QRC:β-CD-K MOFs.

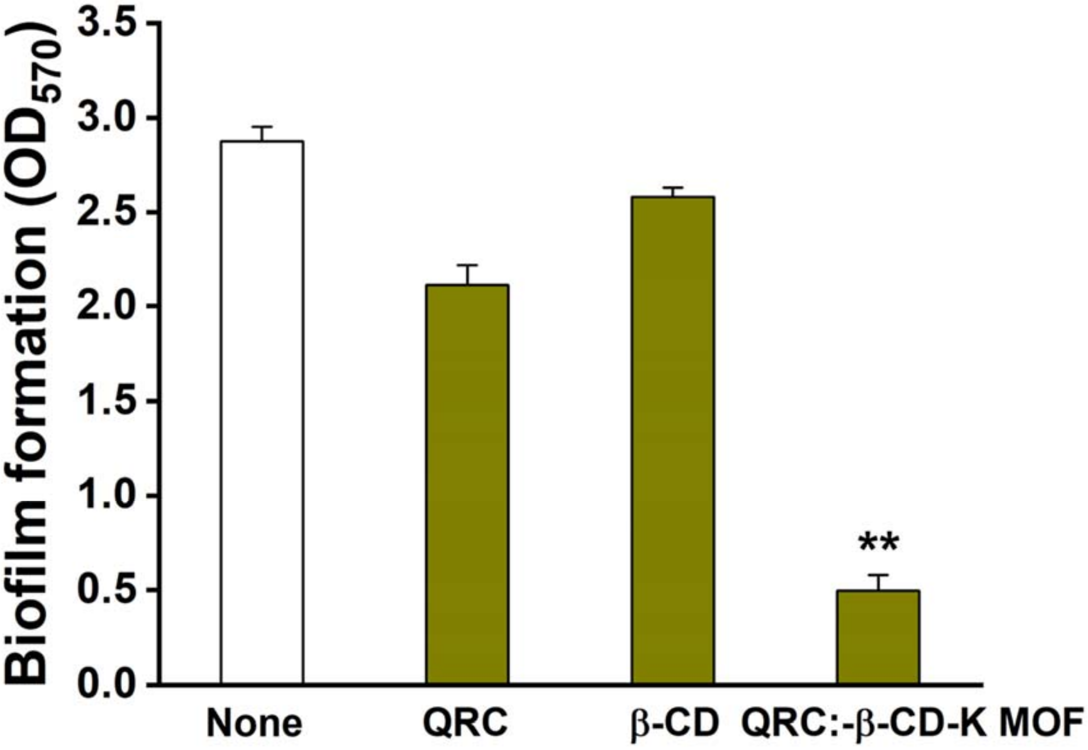

Figure S7. Evaluation of antibiofilm potency of QRC, β-CD and QRC:β-CD-K MOFs at 50 μg/mL concentrations against *C. albicans* DAY 185.

Table S1. Antifungal efficacy of QRC:β-CD-K MOFs at 0, 250, 500, and 1000 μg/mL concentrations against *C. albicans* DAY 185.

**Name of the fungal strain**	**Zone of Inhibition (mm)**			
	**None**	**250 μg/mL**	**500 μg/mL**	**1000 μg/mL**
***C. albicans* DAY 185**	6.0 ± 0.1	17.0 ± 0.9	22.0 ± 1.1	28.0 ± 1.2
